# Effects of phthalates on normal human breast cells co-cultured with different fibroblasts

**DOI:** 10.1371/journal.pone.0199596

**Published:** 2018-06-25

**Authors:** Fang-Ping Chen, Mei-Hua Chien, Huang-Yang Chen, Yuet-Tong Ng

**Affiliations:** 1 Department of Obstetrics and Gynecology, Chang Gung Memorial Hospital, Keelung, Taiwan; 2 Department of Medicine, College of Medicine, Chang Gung University, Kwei-Shan, Taoyuan, Taiwan; 3 Department of General Surgery, Chang Gung Memorial Hospital, Keelung, Taiwan; 4 Department of Anesthesiology, Chang Gung Memorial Hospital, Keelung, Taiwan; University of Bergen, NORWAY

## Abstract

Whether or not phthalates play a role in breast carcinogenesis remains to be determined. The goal of this study was to explore the effects of phthalates on the growth of normal MCF-10A breast cells modulated by breast fibroblasts. Fibroblasts were derived from normal mammary tissue adjacent to both estrogen receptor (ER) positive and negative primary breast cancers, which were grown separately from nontumorigenic MCF-10A epithelial cells. MCF-10A co-culture cells were treated with 10 nM 17β-estradiol (E_2_), Butyl benzyl phthalate (BBP), di(n-butyl) phthalate (DBP), and di(20ethylhexyl) phthalate (DEHP) (10 and 100 nM). After incubation for 120 hours, the cells were harvested and extracted for MTT assay. Western blot analysis was used to evaluate the proliferative pathway proteins and the effects on ER α. Only fibroblasts from ER (+) breast cancer significantly stimulated proliferation of MCF-10A cells. Exposure of the co-culture to E_2_, BBP, DBP, DEHP, and E_2_ combined with one of these phthalates resulted in significantly increased cell proliferation, as well as proliferating cell nuclear antigen (PCNA) and ER α expressions. The present study demonstrates that phthalates express a significant influence in fibroblast–epithelial interactions, similarly to the effects of E_2_ on breast cells. The effects of phthalates on normal breast cells depend upon ER modulating actions. In breast carcinogenesis, phthalates should be considered as having endocrine disrupting potential, even at low concentrations.

## Introduction

It is generally recognized that phthalates are endocrine disruptors. Epidemiological studies have demonstrated that exposure to diethyl phthalate in the environment may increase the risk of breast cancer [1]. A Canadian case-control study also noted that women working in the automotive and food-canning industries have a fivefold increased risk for premenopausal breast cancer, suspected to be related to their phthalates exposure [2]. Therefore, the role of phthalates, as endocrine disruptors, in steroid hormone-dependent cancers, such as breast cancer, has been strongly debated.

The association between phthalates exposure and the risk of breast cancer is still under contention. Besides the aformentioned epidemiological evidences, several in vitro studies have also demonstrated that phthalates are associated with increased breast cancer risk [3–5]. Our previous studies revealed that even at a very low concentration (10nM), BBP, DBP, and DEHP were not only capable of inducing a proliferative effect on breast cancer cells through the PI3K/Akt signaling pathway but also exhibiting estrogenic activity and additive effects when combined with 17β-estradiol [6, 7]. Although the aforementioned results revealed a strong possible association between phthalates and breast cancer risk, those studies assessing the effects of phthalates have concentrated on established breast cancers. If phthalates have a potential role during breast carcinogenesis, theoretically they should promote the growth of epithelial cells derived from benign breast disease, such as MCF-10A cells.

Normal breast development is regulated by dynamic interactions between breast epithelial cells and their associated stroma. It is also suggested that the fibroblast-epithelial interactions are possibly equally important during breast cancer progression [8–10]. However, during breast carcinogenesis, the effect of fibroblasts on the growth of epithelial cells derived from benign breast disease, not breast cancer cells, should be evaluated.

Toxicological evidence has shown that BBP, DBP, and DEHP may alter or mimic estradiol (E2) in vivo and in vitro [4, 11]. However, whether or not these phthalates at the present reference doses play a role in breast carcinogenesis remains to be determined. The goal of this study was to establish a co-culture system of MCF-10A cells and primary human fibroblasts from ER (+) and ER (-) breast cancer so as to study the effects of phthalates on normal breast cells.

## Materials and methods

### Ethics

This study was approved by the Institutional Review Board of Chang Gung Medical Foundation (IRB 103-6947B and IRB 104-8776B), the Clinical Monitoring Research Program (Chang Gung Memorial Hospital, Keelung) (CMRPG2E0361), and National Science Council, Taiwan (NMRPD1F1231). We started this research study on January 1, 2016. This study was undertaken with the understanding and appropriate informed consent of each participant with written documentation.

### Reagents

Phthalates (10 and 100 nM) including butyl benzyl phthalate (BBP), di(n-butyl) phthalate (DBP), and di(20ethylhexyl) phthalate (DEHP)were purchased from SUPEL Co. (Bellefonte, PA, USA). 17β-estradiol (E2, 10 nM) was purchased from Sigma Chemical Co. (St. Louis, MO, USA). The compounds were reconstituted according to the manufacturer’s instructions as stated on the package insert and were stored in aliquots at -20°C.

### Cell culture

#### A. MCF-10A cells

MCF-10A cells were obtained from the American Type Culture Collection (ATCC, Manassas, VA, USA). The MCF-10A normal mammary epithelial cell line was cultured in Dulbecco modified Eagle medium/F12 (1:1) media containing 10% fetal bovine serum, 20 ng/mL epidermal growth factor, 0.5 mg/mL hydrocortisone, 100 ng/mL cholera toxin, 10 μg/mL insulin, 2 mM L-glutamine, 100 U/mL penicillin, and 100 mg/mL streptomycin in humidified air (5% CO_2_) at 37°C.

#### B. Isolation of primary fibroblast

After obtaining informed consent for participation, consecutive breast tissue samples were obtained from fresh surgical tissue at the time of mastectomy procedures at Keelung Chang Gung Memorial Hospital. Histological assessment and tumor typing were performed at the Department of Pathology at Keelung Chang Gung Memorial hospital. Three ER (+) and three ER (-) stromal cells were collected from six women. Data related to the patients’ characteristics, sample size, and type of surgery, were recorded. According to procedures as described by Proia and Kuperwasser [12] with minor modifications, breast tissue was carefully dissected under a stereo-microscope to exclude as much adipose tissues as possible, and then cut with fine scissors to 3–5 mm^3^. 1–2 g of tissue was subsequently transferred to a 15-ml conical polypropylene tube filled with 10 ml of working collagenase solution and incubated on a rotator at 37°C until the large tissue fragments were dissociated. The tubes were removed from the incubator and were left to stand for 2–5 minutes to allow the organoids to settle. The supernatant was decanted to a fresh tube and then washed with PBS and centrifuged (300*g* on a tabletop centrifuge at 4°C) for 5 minutes. The washing and centrifuging was repeated three or more times with 10 ml of PBS. The fibroblastic nature of the isolated cells was confirmed through microscopic determination of the morphology portrait and immunofluorescence characterization using antibodies against the fibroblast surface protein (FSP) (Abcam, Cambridge, MA, USA). The primary fibroblast cells were maintained in DMEM/F12, supplemented with 10% fetal bovine serum, 1.5mg/L sodium bicarbonate and 100 IU/ml penicillin with 100 g/ml streptomycin and cultured at 37°C in a humidified 5% CO_2_ environment.

#### C. Co-culture system

For construction of the cell-cell interactive environment, MCF-10A cells were plated in a 6-well plate at a density of 4 × 10^5^ cells/well. Contrastingly, primary fibroblasts were plated with 4 × 10^5^ cells/well in the 0.4 μm pore size of the 6-transwell insertion (Corning, NY, USA). Co-culture was continuously cultured for 120 hrs. The co-cultures were incubated with serum-free DMEM /F12 1.5mg/L sodium bicarbonate, 100 IU/ml penicillin and 100 μg/ml streptomycin, and were also treated separately with phthalates (BBP, DBP, and DEHP, 10 and 100 nM), estradiol (E2, 10 nM) alone, and a combination of estradiol and phthalates.

### Evaluation

#### A. Cell proliferation assay (MTT assay)

Cell viability was determined using a 3-(4,5-Dimethylthiazol-2-yl)-2, 5-diphenyltetrazolium bromide (MTT) assay (Sigma Chemical Co, St. Louis, MO, USA) [13]. The absorbance of blue formazan crystals was measured at 570 nm using a spectrophotometer (Molecular Device Spectramax M3, Sunnyvale, CA, USA). The quantity of the formazan product was directly proportional to the number of viable cells in the culture medium.

#### B. Protein extraction and western blot analysis

The cell pellets were lysed for 30 min in lysis buffer (50 mM Tris, pH 7.5, 0.5 M NaCl, 1.0 mM EDTA, pH 7.5, 10% glycerol, 1 mM basal medium Eagle, 1% Igepal-630, and proteinase inhibitor cocktail tablet) (Roche Applied Science, IN, USA) and then centrifuged at 12 000 ***g*** for 10 min. The supernatants were removed and placed in new Eppendorf tubes for western blot analysis. To avoid probable loss of antigen during stripping and re-probing of western blot membranes, which may possibly lead to erroneous measurements [14], the internal control (β-actin) and protein of interest were probed separately in duplicate blots. The same amount of protein extracted from the MCF-10A cells was loaded in duplicate in 12% gradient SDS–PAGE and transferred onto nitrocellulose membranes. Nonspecific protein binding was blocked using a blocking buffer at RT for 1 hour (5% milk, 20 mM Tris–HCl, pH 7.6, 150 mM NaCl, and 0.1% Tween 20). The membranes were blotted with proliferating cell nuclear antigen (PCNA) (Chemicon, Temecula, CA, USA), phosphorylated estrogen receptor (ER) α (Upstate Biotechnology, Lake Placid, NY,USA) and β-actin (Thermo, Rockford, IL, USA) antibodies and incubated in 4°C blocking buffer overnight. Densitometric analysis of immunoblots was performed using the Bio Rad molecular imager versadoc MP 4000 system (Bio Rad, Hercules, CA, USA). Western blotting was performed in triplicate for each MCF10A-breast cancer fibroblast pair. However, we also performed an additional western blot analysis, as per the reviewer’s suggestion, with the membrane being first blotted with the PCNA antibody and then stripped and re-probed with the β-actin antibody on MCF-10A cells co-cultured with fibroblast from normal mammary tissue adjacent to ER (+) primary breast cancers. Similar results were obtained as when PCNA and β-actin were run on the same gel (S1 Fig).

### Statistical analysis

Data for cell proliferation were expressed using percents and then compared with vehicle-treated control cells, which were arbitrarily assigned 100%. All data was measured versus controls from three ER (+) and three ER (-) patients respectively. Statistical significance of difference was calculated using Student’s *t*-test for paired data with the level of significance selected at *p* ≦ 0.05. All data was analyzed using Excel statistical software.

## Results

### Effects of phthalates on cell viability of MCF-10A normal breast cells

**Fig 1:**
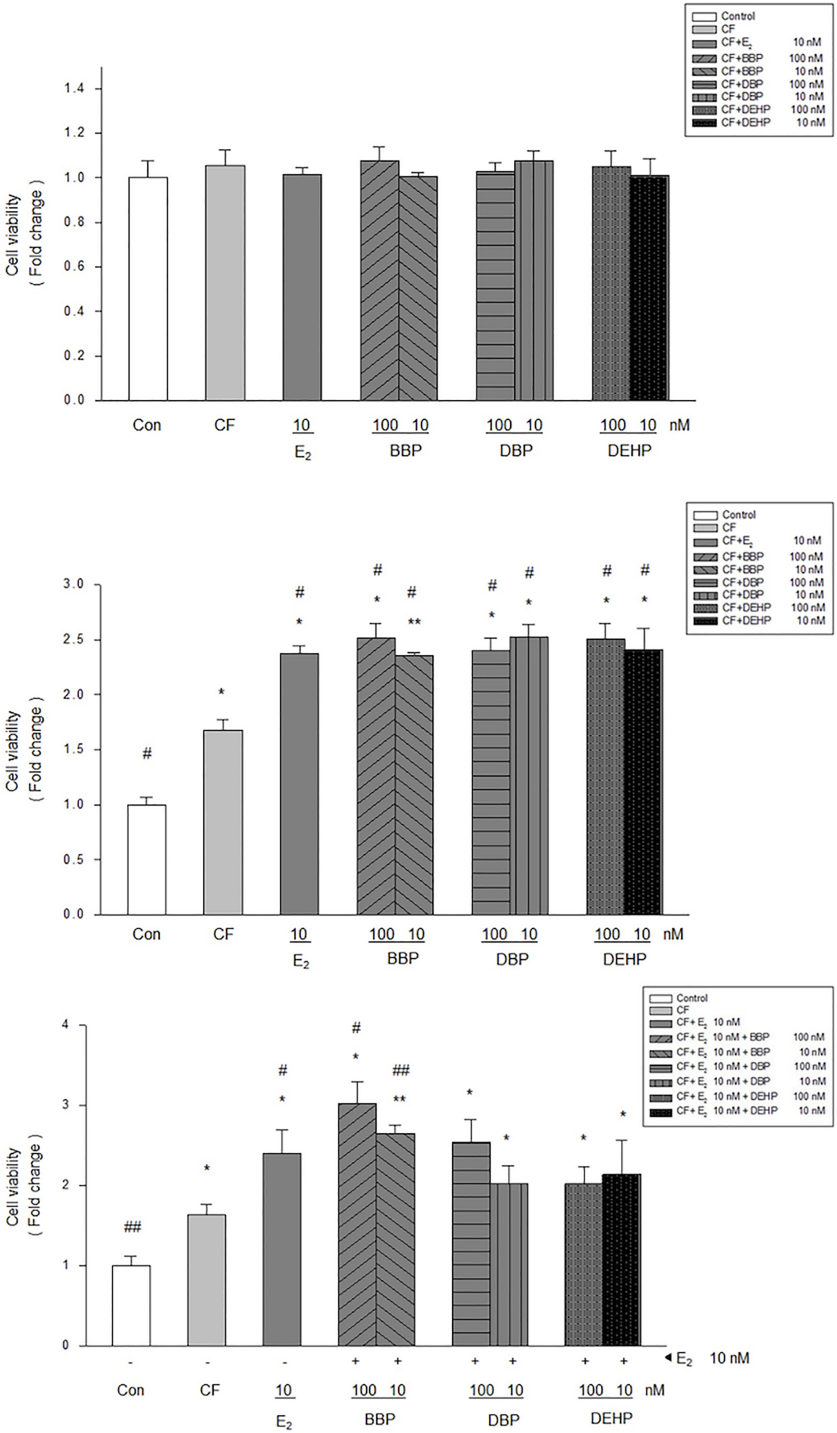
Effects of phthalates and estradiol on viability of MCF-10A cells in co-cultures with fibroblasts from different breast cancers. Phthalates (BBP, DBP, and DEHP, 10 and 100 nM) and estradiol (E2, 10 nM) induced different effects on cell viability of MCF-10A in co-cultures with fibroblasts from normal mammary tissue adjacent to estrogen receptor negative (A) or positive (B) primary breast cancers. (C) Phthalates (BBP, DBP, and DEHP, 10 and 100 nM) combined with E_2_ did not demonstrate an additive effect on the growth of MCF-10A co-culture with fibroblasts from normal mammary tissue adjacent to estrogen receptor positive primary breast cancers. Con: control (MCF-10A alone), CF: control fibroblast (MCF-10A co-cultured with fibroblast), *: P<0.05 vs. control, **: P<0.001 vs. control, #: P<0.05 vs. CF,. ##: P<0.001 vs. CF.

As shown in Fig 1A, fibroblasts, derived from normal mammary tissue adjacent to estrogen receptor (ER) negative primary breast cancers, did not have an effect on the growth of MCF-10A cells. E_2_, BBP, DBP, and DEHP also revealed no effects on MCF-10A cells in this co-culture. In contrast, fibroblasts from normal mammary tissue adjacent to ER (+) primary breast cancers significantly increased MCF-10A proliferation. Furthermore, E_2_, BBP, DBP, and DEHP substantially stimulated MCF-10A cells in this co-culture system, but the effects did not change with increased concentration of these three phthalates (Fig 1B). As seen in Fig 1C, BBP, DBP, or DEHP combined with E_2_ did not demonstrate an additive effect on the growth of MCF-10A co-culture. To avoid results that reflect a cell line-based bias, we used another normal human mammary epithelial cell line (H184B5F5/M10) (BCRC60197, Bioresource Collection and Research Center, Hsinchu, Taiwan) for the aforementioned experiments. E_2_, BBP, DBP, and DEHP also revealed similar results as those that used the MCF-10A cell line (Parts A and B in S2 Fig).

### Proliferative effects of phthalates in MCF-10A cells co-cultured with fibroblasts

**Fig 2:**
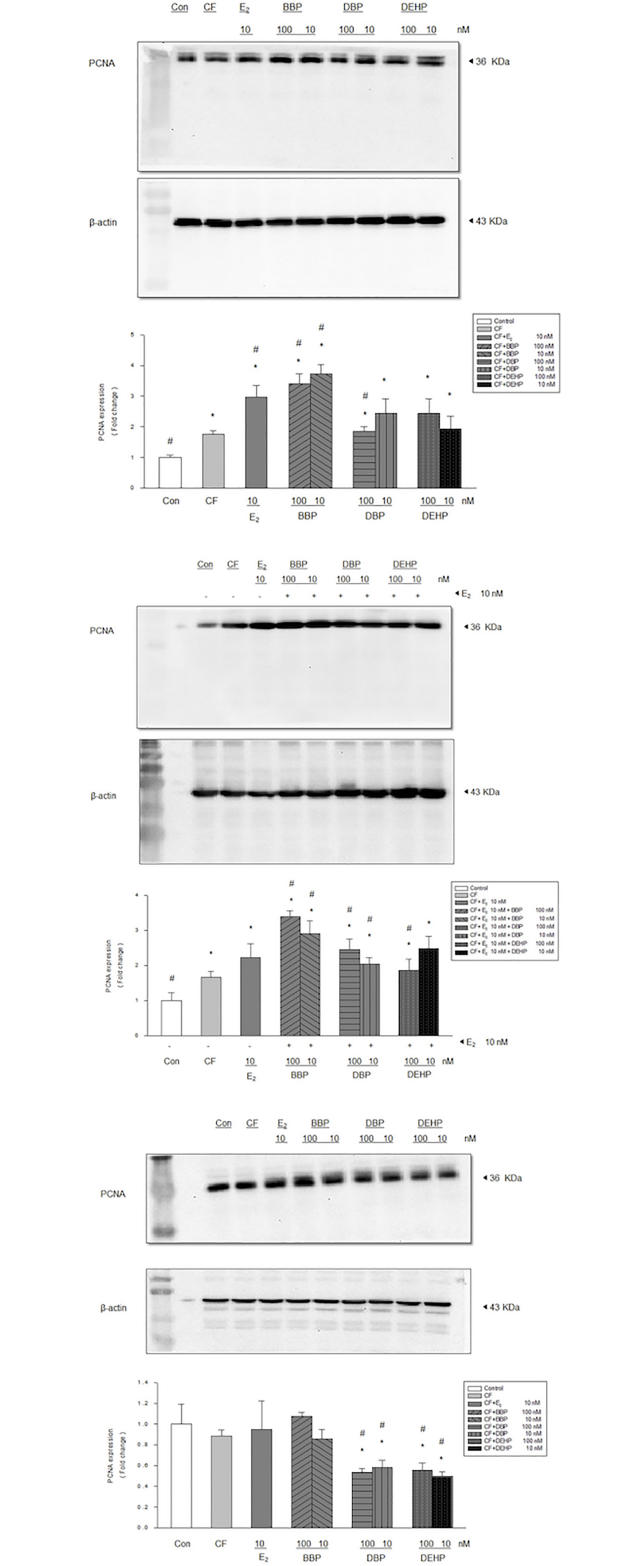
Effects of phthalates and estradiol on PCNA expression of MCF-10A in co-cultures with fibroblasts from different breast cancers. PCNA expression was increased in MCF-10A co-cultured with fibroblasts from ER (+) primary breast cancer treated by (A) Phthalates, estradiol (E2) or (B) a combination of E_2_ and phthalates, (C) but not in MCF-10A co-cultured with fibroblasts from ER (-) primary breast cancers. Con: control (MCF-10A alone), CF: control fibroblast (MCF-10A co-cultured with fibroblast),*: P<0.05 vs. control, #: P<0.05 vs. CF.

We further verified the activities of E_2_, BBP, DBP, and DEHP on MCF-10A cells co-cultured with fibroblast from normal mammary tissue adjacent to ER (+) primary breast cancers. PCNA was significantly increased in cultures with DEHP, BBP, and DBP (at concentrations of 10 nM and 100 nM) as compared with MCF-10A co-cultured both with and without fibroblasts (Fig 2A). E_2_ (10 nM) enacted similar effects on MCF-10A as that of phthalates. Although DEHP, BBP, and DBP (at concentrations of 10 nM and 100 nM) combined with E_2_ (10 nM) significantly induced PCNA expression, no additive effects were noted (Fig 2B). As with MCF-10A cells co-cultured with fibroblast from normal mammary tissue adjacent to ER(-) primary breast cancers, both DBP and DEHP (10 and 100 nM) significantly decreased PCNA expression, while BBP and E2 did not have any effect on PCNA expression (Fig 2C). Further analysis using another mammary epithelial cell line (H184B5F5/M10) co-cultured with fibroblast from both normal mammary tissue adjacent to both ER(-) (Part A in S3 Fig) and ER(+) (Part B in S3 Fig) primary breast cancers was also performed, which revealed similar results as the co-cultured MCF-10A cells. In the H184B5F5/M10 co-cultured with ER(-) fibroblasts, neither E2 nor any of the phthalates, including BBP, DBP, and DEHP, exhibited any effect on PCNA expression. As for the H184B5F5/M10 co-cultured with ER(+) fibroblasts, similar stimulatory effects on PCNA by E2, BBP, and DBP were also noted.

### Effects of phthalates on ER α in MCF-10A cells co-cultured with fibroblasts

**Fig 3:**
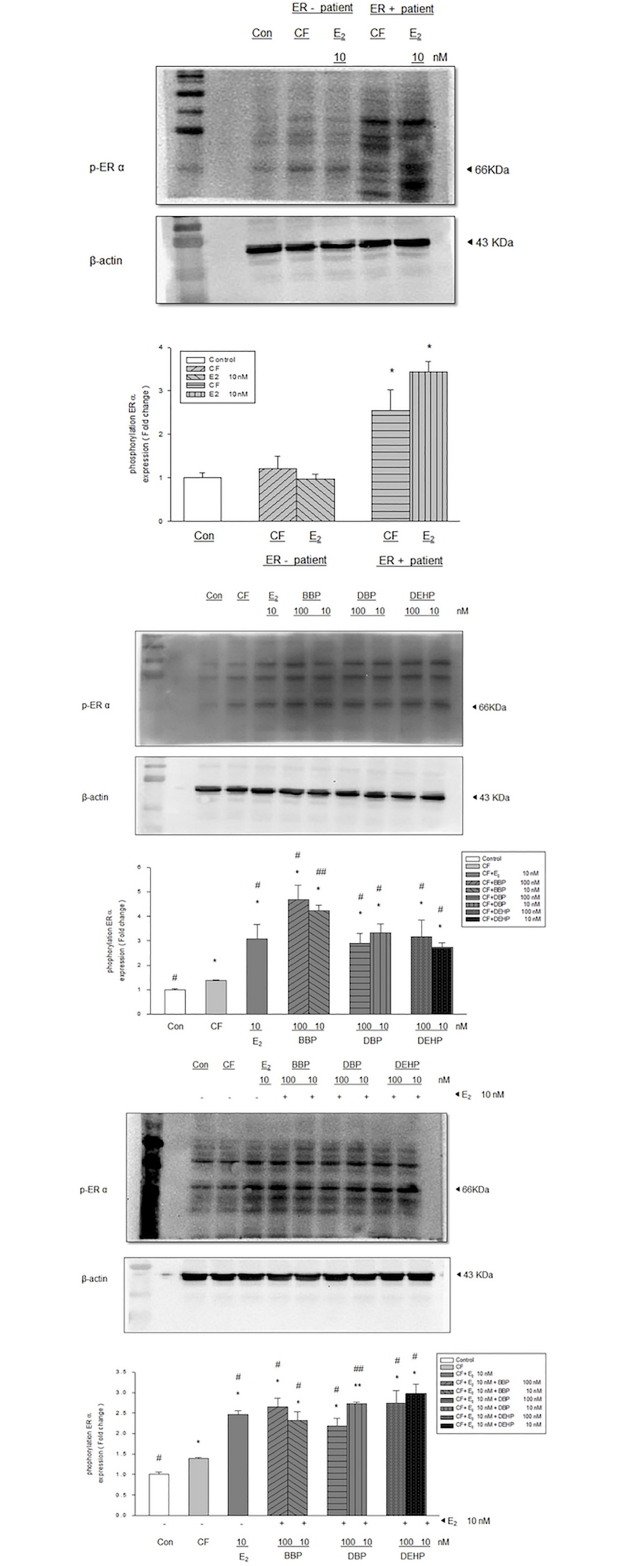
Effects of phthalates and estradiol on ER α expression in MCF-10A cells co-cultured with fibroblasts. (A) The expression of ER α phosphorylation was only noted in MCF-10A co-cultured with fibroblasts from ER (+) breast cancers, but not in MCF-10A cells alone or co-cultured with fibroblasts from ER (-) breast cancers. ER α expression in MCF-10A cells co-cultured with ER (+) fibroblasts was significantly increased by phthalates (BBP, DBP, DEHP) and estradiol (E2) alone (B), as well as with a combination of E_2_ and phthalates (C). Con: control (MCF-10A alone), CF: control fibroblast (MCF-10A co-cultured with fibroblast), *: P<0.05 vs. control, **: P<0.001 vs. control, #: P<0.05 vs. CF, ##: P<0.001 vs. CF.

As shown in Fig 3A, the expression of ER α phosphorylation in MCF-10A cells alone or co-cultured with fibroblasts from ER (-) breast cancers showed negligible expression as compared with that of MCF-10A co-cultured with fibroblasts from ER (+) breast cancers. E_2_ (10 nM) induced significant ER α expression in MCF-10A co-cultured with fibroblasts from ER (+) breast cancers, but not in co-culture with fibroblasts from ER (-) breast cancers. BBP, DBP, and DEHP (at concentrations of 10 nM and 100 nM) combined without (Fig 3B) and with (Fig 3C) E_2_ (10 nM), just like E_2_ (10 nM), notably induced the expression of ER α phosphorylation in MCF-10A cells, which were co-cultured with fibroblasts from ER(+) primary breast cancers. However, the combination of one of the phthalates with E_2_ did not change ER α phosphorylation expression in MCF-10A cells.

## Discussion

The present study demonstrates that BBP, DBP, and DEHP are all endocrine disruptors and may induce normal breast cell proliferation, even at low concentrations. In addition, we confirm the possible effects of stromal-epithelial interactions, in which fibroblast from surrounding tissue of ER (+) breast cancers, but not those from ER (-) breast cancers, may stimulate normal breast cell proliferation by inducing the expression of ER α. As the stromal environment changes, BBP, DBP, and DEHP may have potential for breast cancer development.

Our previous study [15] and other reports [16, 17] have demonstrated that MCF-10A, like most normal breast epithelial cells, does not express significant level of ER, and is negatively regulated by the presence of E_2_. It has been proven that E_2_-induced proliferation in normal breast cells is highly regulated by paracrine mechanisms [17,18]. Thus, in order to evaluate the carcinogenesis of xenoestrogens, co-culture of MCF-10A with stromal fibroblasts surrounding breast cancers was considered to represent pre-invasive breast disease.

There are varying reports regarding the proliferative response of normal mammary epithelial cells in the presence of breast carcinoma-associated fibroblasts [19, 20]. It may be related to the differences in ratio of epithelial cells to fibroblasts, in which a predominance of fibroblast correlates with an increase in growth of co-cultured breast epithelial cells. Thus, we used an epithelial cell: fibroblast ratio of 1:1. However, fibroblasts from different types of breast cancer were not considered in these previous studies. As compared to fibroblasts from normal breast tissue, fibroblasts from breast cancer have been reported to express increased amounts of specific extracellular matrix molecules, various molecules that modulate the extracellular matrix, and several peptide growth factors [10, 21]. It is possible that fibroblasts from ER (+) and ER (-) breast cancer may also have differing contents. The present study is the first to evaluate the various responses of normal breast cells to fibroblasts from ER (+) and ER (-) breast cancers. We found that fibroblasts from ER (+) breast cancers, but not those from ER (-) breast cancers, induced significant growth of MCF-10A breast cells. Aside from the difference of ER α expression induced by various original fibroblasts, the role of fibroblasts from ER (-) breast cancer on breast carcinogenesis remains to be determined.

Although phthalates have a lower potency of estrogenic activity as compared with endogenous steroid estrogens and are present in low levels in human tissues, epidemiological evidence has revealed a high probability of the association between phthalates and breast carcinogenesis [1, 2]. In addition, the U.S. Center for Disease Control and Prevention has stated that human health effects from exposure to low levels of phthalates are unknown. Therefore, the significance of phthalates at their present reference doses remains to be determined. Our previous study demonstrated that even at a low concentration (10 nM), BBP, DBP, and DEHP not only induced a proliferative effect, but also exhibited estrogenic effects, on breast cancer cells [11]. It has been reported that there is a correlation between increased Akt activity and human breast epithelial stem cells that have been exposed to a higher concentration (10^-6^M) of BBP and DBP [5]. The present study further confirmed that even at low concentrations (10 and 100 nM), phthalates induced significant Akt activity, proliferation, and ER α expression through increased ER α affinity in MCF-10A co-culture with fibroblasts from ER (+) breast cancer, but not in ER (-) breast cancer. It also revealed that the effect of phthalates on normal breast cells depends upon ER’s modulating action. Furthermore, the effects of phthalates on normal breast cells were similar to those of E_2_, which confirms that phthalates are endocrine disruptors even at low levels.

There is increasing concern that phthalates may interfere with endogenous estrogens. Rajapakse et al. showed that weak xenoestrogens could significantly modulate the effects of 17β-estradiol, even when each compound was present below the concentration at which no effect was observed [22]. However, the present study revealed that estradiol combined with phthalates at low concentrations did not induce an additive effect. Further investigation is needed to determine whether this is attributed to too low levels of phthalates, which cannot counteract the strong estrogenic effects of E_2_.

Our study has an important limitation. It has been reproted that the assessment of BBP, DBP, and DEHP on uterine wet weight and vaginal cell cornification using ovariectomized Sprague-Dawley rats did not extract any in vivo estrogenic response [23]. Although the present study demonstrated that the effects of BBP, DBP, and DEHP on MCF-10A normal breast cells may be related to estrogenic activity, it does not ensure similar estrogenic effects in vivo, especially at such low concentrations. Additional studies to assess the estrogenic effects of phthalates on normal animal breast cells are required. However, BBP, DBP, and DEHP are used in a wide variety of products, such as toys, food packaging, pharmaceuticals, blood bags and tubing, and some cosmetics and personal care products. Therefore, the carcinogenic effects of phthalates should not be ignored or misconstrued as insignificant solely due to the low concentrations found in the human body or their lower potency as compared with endogenous estrogens.

## Supporting information

S1 Fig Effects of phthalates and estradiol on PCNA expression of MCF-10A in co-cultures with fibroblasts from ER (+) breast cancers. PCNA expression was increased in MCF-10A co-cultured with fibroblasts from ER (+) primary breast cancer treated by phthalates and Estradiol (E_2_). Con: control (MCF-10A alone), CF: control fibroblast (MCF-10A co-cultured with fibroblast),*: P<0.05 vs. control, #: P<0.05 vs. CF.
(PDF)

S2 Fig Effects of phthalates and estradiol on viability of H184B5F5/M10 cells in co-cultures with fibroblasts from different breast cancers. Phthalates and estradiol (E_2_) induced different effects on cell viability of H184B5F/M10 cells in co-cultures with fibroblasts from estrogen receptor negative (A) or positive (B) breast cancers. Con: control (MCF-10A alone), CF: control fibroblast (MCF-10A co-cultured with fibroblast), *: P<0.05 vs. control, #: P<0.05 vs. CF.
(PDF)

S3 Fig Effects of phthalates and estradiol on PCNA expression of H184B5F5/M10 cells in co-cultures with fibroblasts from different breast cancers. Phthalates and estradiol (E_2_) induced different effects on PCNA expression in H184B5F5/M10 cells co-culture with fibroblast from estrogen receptor negative (A) or positive (B) breast cancer. Con: control (MCF-10A alone), CF: control fibroblast (MCF-10A co-cultured with fibroblast), *: P<0.05 vs. control, #: P<0.05 vs. CF.
(PDF)
