# Supplementary figures and images for: Effects of phthalates on normal human breast cells co-cultured with different fibroblasts
Source: PLoS One. 2018 Jun 25;13(6):e0199596. doi: 10.1371/journal.pone.0199596 (PMC6016934; doi:10.1371/journal.pone.0199596)

ER + patient

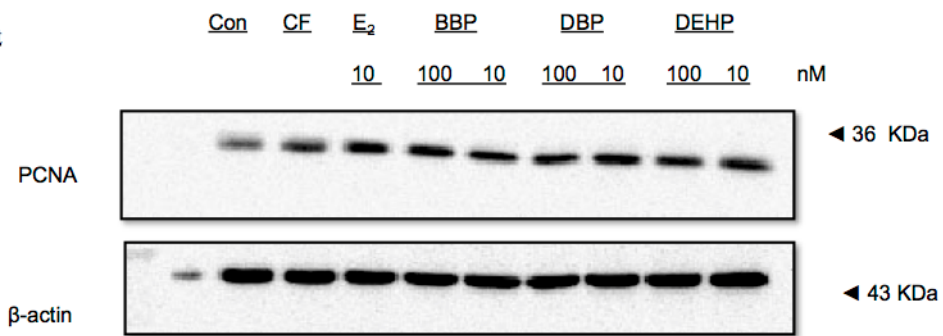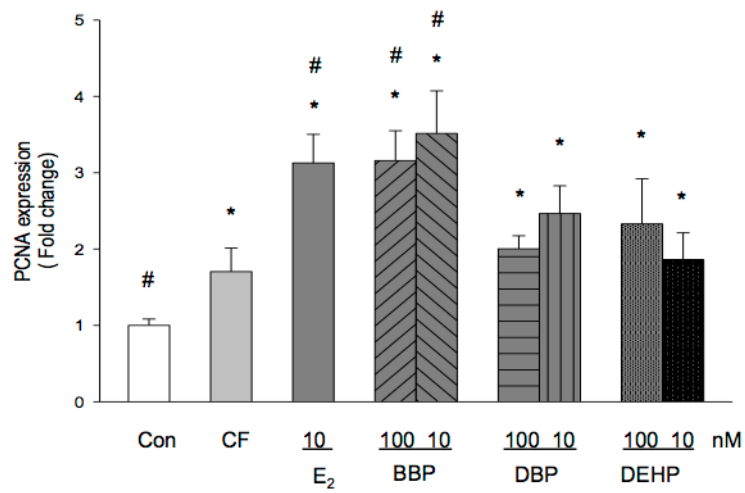

Supplement: S1 Fig — PCNA expression was increased in MCF-10A co-cultured with fibroblasts from ER (+) primary breast cancer treated by phthalates and Estradiol (E2). Con: control (MCF-10A alone), CF: control fibroblast (MCF-10A co-cultured with fibroblast),*: P<0.05 vs. control, #: P<0.05 vs. CF. (PDF) [file pone.0199596.s001.pdf]

Figure A in S2 File

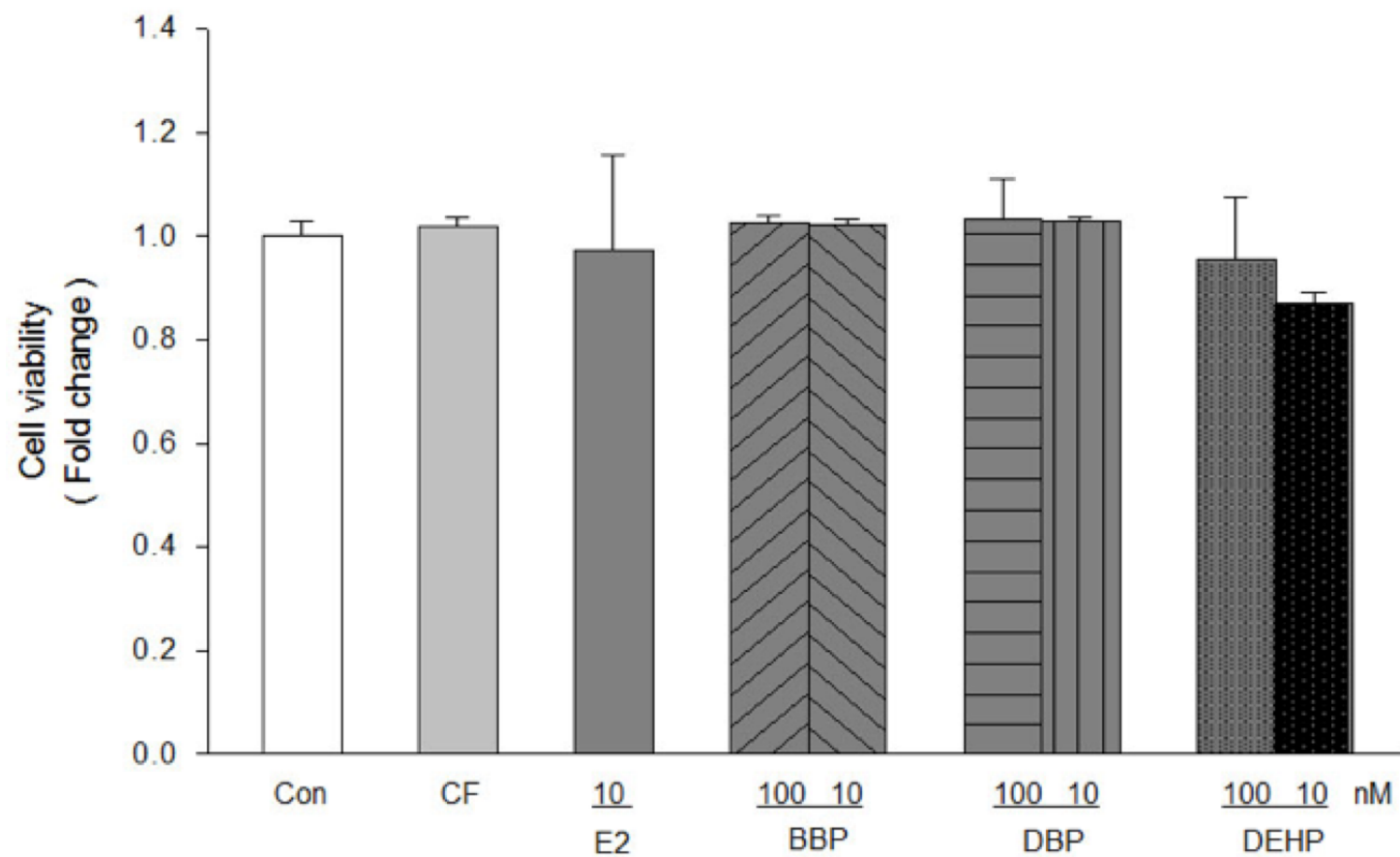

Figure B in S2 File

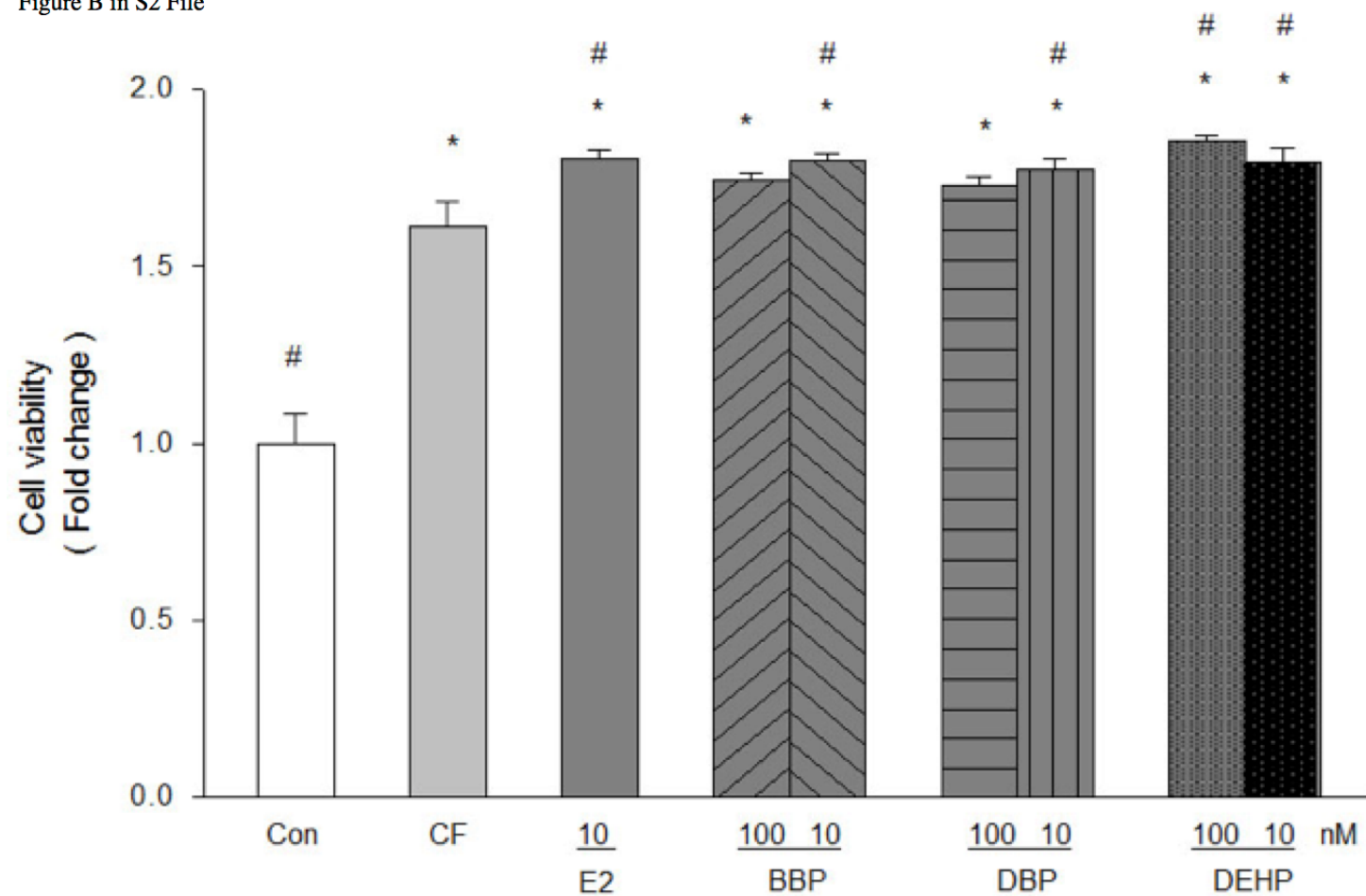

Supplement: S2 Fig — Phthalates and estradiol (E2) induced different effects on cell viability of H184B5F/M10 cells in co-cultures with fibroblasts from estrogen receptor negative (A) or positive (B) breast cancers. Con: control (MCF-10A alone), CF: control fibroblast (MCF-10A co-cultured with fibroblast), *: P<0.05 vs. control, #: P<0.05 vs. CF. (PDF) [file pone.0199596.s002.pdf]

Figure A in S3 File

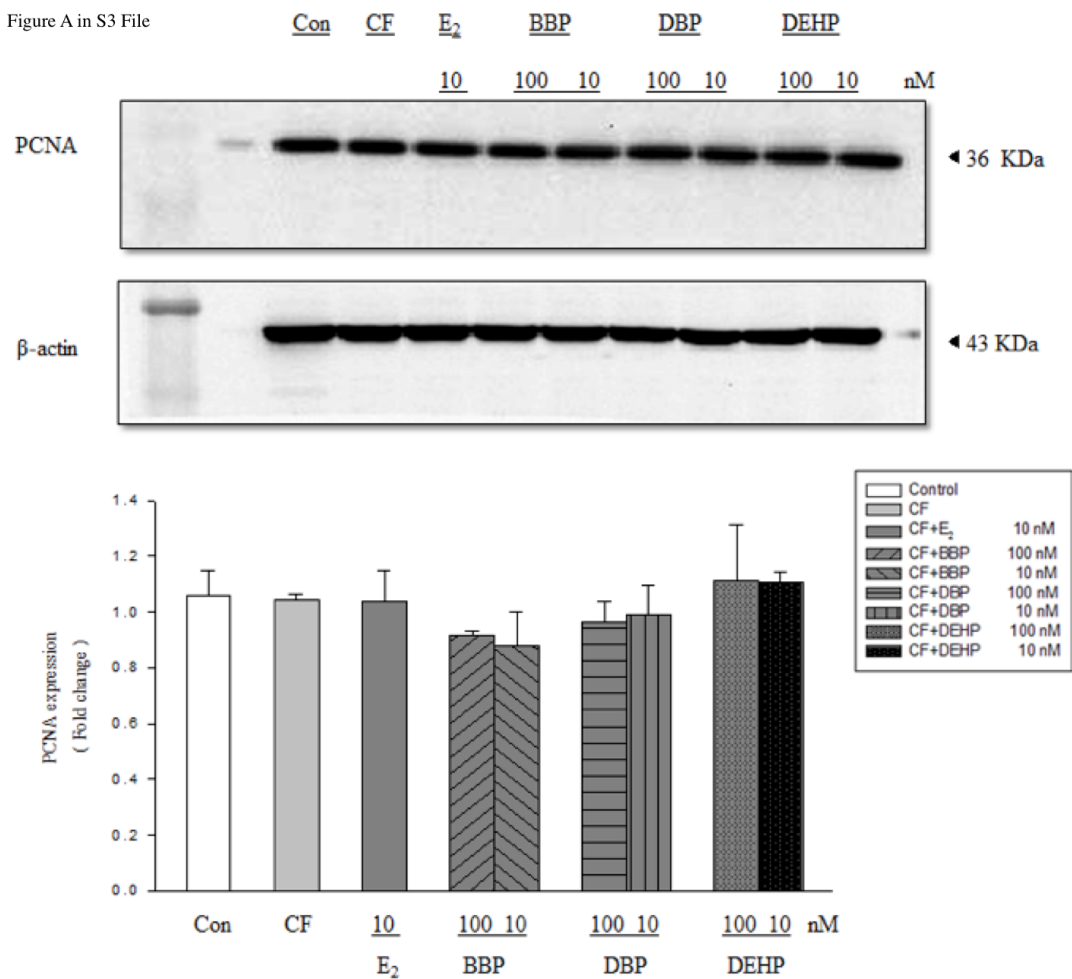

Figure B in S3 File

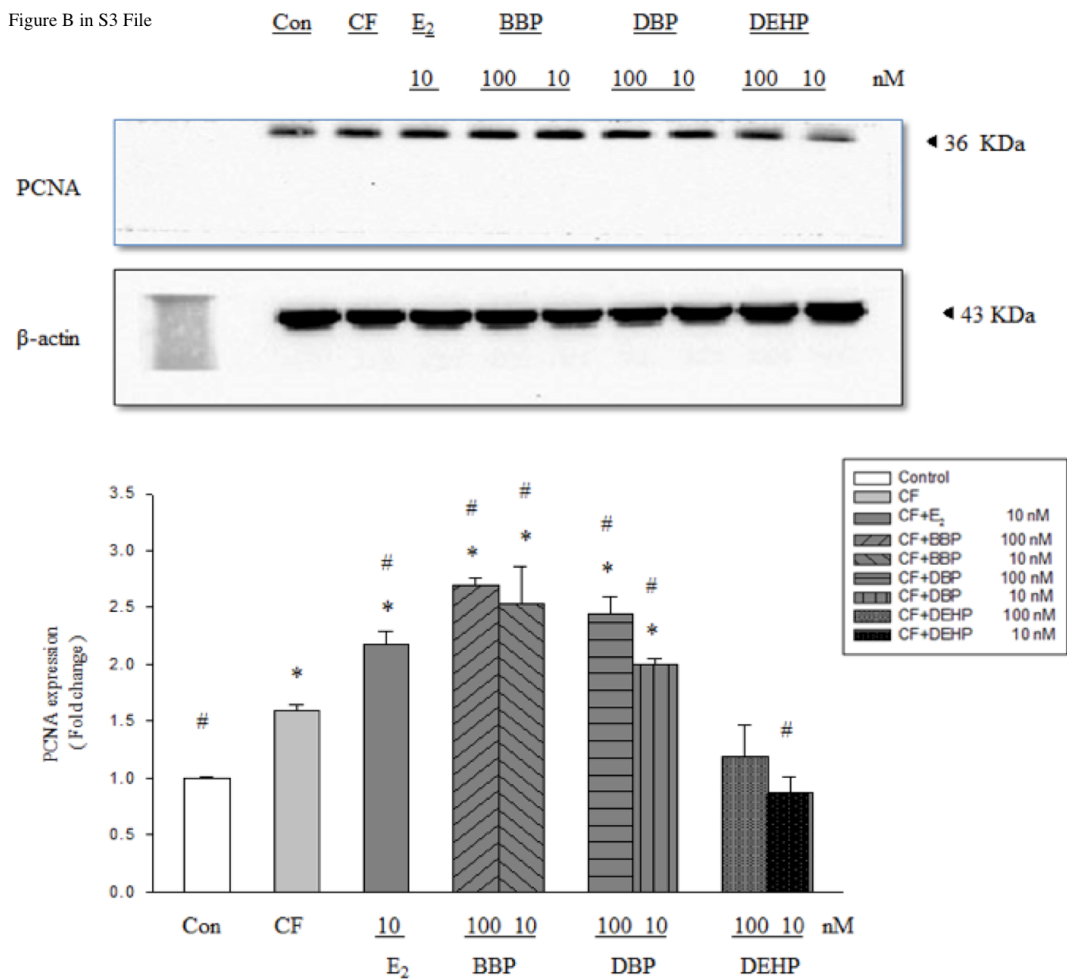

Supplement: S3 Fig — Phthalates and estradiol (E2) induced different effects on PCNA expression in H184B5F5/M10 cells co-culture with fibroblast from estrogen receptor negative (A) or positive (B) breast cancer. Con: control (MCF-10A alone), CF: control fibroblast (MCF-10A co-cultured with fibroblast), *: P<0.05 vs. control, #: P<0.05 vs. CF. (PDF) [file pone.0199596.s003.pdf]
